# COVID-19 vaccination in pregnancy: the impact of multimorbidity and smoking status on vaccine hesitancy, a cohort study of 25,111 women in Wales, UK

**DOI:** 10.1186/s12879-023-08555-8

**Published:** 2023-09-11

**Authors:** Mohamed Mhereeg, Hope Jones, Jonathan Kennedy, Mike Seaborne, Michael Parker, Natasha Kennedy, Ashley Akbari, Luisa Zuccolo, Amaya Azcoaga-Lorenzo, Alisha Davies, Krishnarajah Nirantharakumar, Sinead Brophy

**Affiliations:** 1 National Centre for Population Health and Wellbeing Research, Faculty of Medicine, Health and Life Science, Swansea University Medical School, Swansea, Wales, UK; 2 Data Lab, National Centre for Population Health and Wellbeing Research, Faculty of Medicine, Health and Life Science, Swansea University Medical School, Swansea, Wales, UK; 3 Health Data Research UK, Swansea University Medical School, Swansea, UK; 4 Population Data Science, Faculty of Medicine, Health and Life Science, Swansea University Medical School, Swansea, Wales, UK; 5 Health Data Science Centre, Fondazione Human Technopole, Milan, Italy; 6 School of Medicine, University of St Andrews, Scotland, UK; 7 Hospital Rey Juan Carlos, University of St Andrews, Instituto de Investigación Sanitaria Fundación Jimenez Diaz. Madrid, Madrid, Spain; 8 Research and Evaluation Division, Public Health Wales, Wales, UK; 9 Institute of Applied Health Research, Birmingham University, Birmingham, UK; 10 Administrative Data Research Wales, Swansea University Medical School, Swansea, UK

**Keywords:** COVID-19 vaccination, Pregnancy, Multimorbidity, Smoking status, Vaccine uptake, Vaccine hesitancy, SAIL Databank

## Abstract

**Background:**

Multimorbidity, smoking status, and pregnancy are identified as three risk factors associated with more severe outcomes following a SARS-CoV-2 infection, thus vaccination uptake is crucial for pregnant women living with multimorbidity and a history of smoking. This study aimed to examine the impact of multimorbidity, smoking status, and demographics (age, ethnic group, area of deprivation) on vaccine hesitancy among pregnant women in Wales using electronic health records (EHR) linkage.

**Methods:**

This cohort study utilised routinely collected, individual-level, anonymised population-scale linked data within the Secure Anonymised Information Linkage (SAIL) Databank. Pregnant women were identified from 13th April 2021 to 31st December 2021. Survival analysis was employed to examine and compare the length of time to vaccination uptake in pregnancy by considering multimorbidity, smoking status, as well as depression, diabetes, asthma, and cardiovascular conditions independently. The study also assessed the variation in uptake by multimorbidity, smoking status, and demographics, both jointly and separately for the independent conditions, using hazard ratios (HR) derived from the Cox regression model.

**Results:**

Within the population cohort, 8,203 (32.7%) received at least one dose of the COVID-19 vaccine during pregnancy, with 8,572 (34.1%) remaining unvaccinated throughout the follow-up period, and 8,336 (33.2%) receiving the vaccine postpartum. Women aged 30 years or older were more likely to have the vaccine in pregnancy. Those who had depression were slightly but significantly more likely to have the vaccine compared to those without depression (HR = 1.08, 95% CI 1.03 to 1.14, p = 0.002). Women living with multimorbidity were 1.12 times more likely to have the vaccine compared to those living without multimorbidity (HR = 1.12, 95% CI 1.04 to 1.19, p = 0.001). Vaccine uptakes were significantly lower among both current smokers and former smokers compared to never smokers (HR = 0.87, 95% CI 0.81 to 0.94, p < 0.001 and HR = 0.92, 95% CI 0.85 to 0.98, p = 0.015 respectively). Uptake was also lower among those living in the most deprived areas compared to those living in the most affluent areas (HR = 0.89, 95% CI 0.83 to 0.96, p = 0.002).

**Conclusion:**

Younger women, living without multimorbidity, current and former smokers, and those living in the more deprived areas are less likely to have the vaccine, thus, a targeted approach to vaccinations may be required for these groups. Pregnant individuals living with multimorbidity exhibit a slight but statistically significant reduction in vaccine hesitancy towards COVID-19 during pregnancy.

**Supplementary Information:**

The online version contains supplementary material available at 10.1186/s12879-023-08555-8.

## Background

COVID-19 vaccination is recognised as an effective public health strategy [1]. However, vaccinations are increasingly seen by the general population as unsafe and unnecessary [1]. Different populations may have higher rates of vaccine hesitancy [2]. For instance, due to added concerns regarding the health of the baby and potential side effects of the vaccine, pregnant women may be more likely to be hesitant to receive a vaccine [3]. The lack of information, changes in guidance and recommendations surrounding vaccination in pregnancy during the COVID-19 pandemic resulted in some hesitancy among expectant mothers to receive vaccinations [4]. Although there is growing evidence that COVID-19 vaccine is safe and effective for pregnant women, vaccine hesitancy is still a significant issue [5].

In the UK, there has been limited research on the uptake of COVID-19 vaccine during pregnancy at the population level. However, in Scotland, ongoing pregnancies were identified through extensive electronic health records (EHR) linkages in a national, prospective cohort study. It revealed that pregnant women had significantly lower vaccination rates than the general population, 32.3% in pregnancy compared to 77.4% among all women [6]. In England, at least one dose of vaccination was received by 22.7% of pregnant women who gave birth in August 2021. This soared dramatically to 32.3% of women who gave birth in September and again to 53.7% in December 2021. [7]. In Wales, a mixed methods analysis was conducted using routinely collected linked data accessed within the Secure Anonymised Information Linkage (SAIL) Databank and the Born in Wales Birth Cohort. The findings revealed that despite two out of three pregnant women reporting in the survey their willingness to receive the COVID-19 vaccine, only one in three received the vaccine during pregnancy [5]. Even though coverage has increased overall, pregnant women’s uptake remains lower than the general population of the same age group [7].

Various factors may influence vaccine acceptance or refusal. Multimorbidity is defined as the co-occurrence of two or more long-term health diseases, which could include physical and mental health diseases such as diabetes, depression, asthma, and cardiovascular diseases [8]. Long-term health conditions are those that generally last a year or longer and have a significant effect on a person’s quality of life [9]. The COVID-19 pandemic in the United Kingdom (UK) has impacted pregnant women’ mental health, raising the prevalence of depression by 47%. [10]. Research has found that having pre-existing illnesses or multimorbidity is associated with a lower rate of vaccine refusal [11, 12].

Health related behaviours may influence vaccine acceptance, such as smoking habits, which can be classified into current smoker, former smoker, never smoked, or smoking status unknown. An adult sample from the UK was studied to determine the variations negative attitudes towards vaccinations generally and intentions to vaccinate against COVID-19, specifically according to smoking status [13]. It was revealed that in comparison to never smokers and former smokers, current smokers indicated considerably higher uncertainty of vaccinations and any advantages, were more concerned about negative long-term effects, and were more in favour of natural immunity. Given that a large proportion of smokers come from socioeconomically underprivileged and socially disadvantaged communities, a decreased vaccination rate in this population could exacerbate health inequities [13].

Previous research have explored the factors that influence vaccine hesitancy aimed to develop tailored interventions for the most vulnerable populations. In a study of 1,203 young individuals in the United States between the ages of 18 and 25, vaccine hesitancy was found to be considerably higher in current smokers (including electronic cigarette users) than in non-current smokers (36% vs. 22%) [14]. Furthermore, multivariable regression analysis showed that the odds of current smokers reporting COVID-19 vaccine hesitancy were more than twice as high as those of non-current smokers. [14]. Similarly, the prevalence of COVID-19 vaccine hesitancy in the UK was examined, and it was revealed that younger age groups had greater rates of vaccine hesitancy (26.5% in 16–24-year-olds versus 4.5% in 75 and older) [15]. Additionally, there were differences in vaccine hesitancy between ethnic groups. Black and Pakistanis or Bangladeshis people showed higher rates of vaccine hesitancy (71.8% and 42.3% respectively), whereas White British or Irish people showed the lowest rates of hesitancy (15.6%) [15].

The Office for National Statistics (ONS) investigated COVID-19 vaccine hesitancy utilising data from the Opinions and Lifestyle Survey (OPN) for the period from 23rd June to 18th July 2021. There was a higher rate of vaccine hesitancy (8%) among adults living in the most deprived areas of England (based on the Index of Multiple Deprivation) compared to adults living in the least deprived areas (2%) [16].

The current study aimed to investigate the impact of multimorbidity, smoking status, and demographics (age, ethnic group, area of deprivation) on vaccine hesitancy among pregnant women in Wales using electronic health records (EHR) data linkage. Identifying groups with higher vaccine hesitancy is critical to develop targeted interventions to enhance vaccine uptake rates.

## Methods

### Study design and setting

A cohort study utilising routinely collected individual-level; anonymised population-scale linked data within the Secure Anonymised Information Linkage (SAIL) Databank. Data sources include general practitioners (GP), hospital admissions, national community child health, maternal indicators, and vaccination data sources. All women recorded as being pregnant on or after 13th April 2021, aged 18 years or older, and eligible for COVID-19 vaccination were identified. They were linked to the COVID-19 vaccination data for dates up to and including 31st December 2021. Time to vaccination was measured from the pregnancy start date for all women recorded as being pregnant on or after the study start date. For those who began pregnancy prior to the study period, when vaccination was not yet accessible to them, the time was measured from the study start date, which corresponds to when vaccination became accessible. In the case of women who received the first dose prior to pregnancy, the second dose was considered as the first vaccination received during pregnancy for time measurement.

### Data sources and linkage

Analysis was undertaken using anonymised population-scale, individual-level linked routinely collected national-scale data available in the SAIL Databank [17, 18], which anonymously links a wide range of person-based data employing a unique personal identifier. The linkage includes primary care data from Wales Longitudinal General Practice (WLGP) linked with secondary care data from inpatient hospital admissions, inpatient from Patient Episode Database for Wales (PEDW), and outpatient from Outpatient Database for Wales (OPDW), pregnancy and maternity related data from the National Community Child Health Database (NCCHD) and Maternal Indicators (MIDS) and vaccination data from the COVID-19 Vaccination Dataset (CVVD) [5]. The primary care data utilises Read codes, which are predominantly 5-digit codes that relate to diagnosis, medication, and process of care codes. The secondary care data uses International Classification of Diseases version 10 (ICD-10) codes for diagnosis and OPCS Classification of Interventions and Procedures version 4 (OPCS-4) surgical interventions. The NCCHD comprises information pertaining to birth registration, monitoring of child health examinations, and immunisations. The MIDS data contains data relating to the woman at initial assessment and to the mother and baby (or babies) for all births [5]. In addition to these data sources, the Welsh Demographic Service Dataset (WDSD) was linked to extract Lower-layer Super Output Area (LSOA) version 2011 information associated with area-level deprivation. In particular, the Welsh Index for Multiple Deprivation (WIMD) version 2019 was employed as a proxy to assess socioeconomic status [5]. These records were linked at the individual level for all women known to be pregnant in Wales between 13th April 2021 and 31st December 2021. Linkage quality has been assessed and reported as 99.9% for WLGP records and 99.3% for PEDW records [19]. All linkage was at the individual level.

### Study population and key dates

Pregnant women were identified as any woman who had pregnancy codes in the WLGP data or in hospital admissions (PEDW) for pregnancy. Additionally, any mothers recorded in the NCCHD or MIDS data with the baby birth date (referred to as the pregnancy end date) and gestational age at birth available were also identified. The baby’s birth date and gestational age enabled the start date of pregnancy to be determined for those who gave birth during the study period [5]. Data collected included vaccination data, maternal age, ethnic group, WIMD 2019, smoking status, depression, diabetes, asthma, and different types of cardiovascular disease including myocardial infarction, cerebral infarction, and non-haemorrhagic or non-infarction stroke. The WIMD 2019 is an official measure for the relative deprivation of areas of Wales. It combines eight separate domains of deprivation, each compiled from a range of different indicators (income, employment, health, education, access to services, housing, community safety, and physical environment) into a single score and is widely used to measure deprivation in Wales [5]. Ethnic groups are categorised in SAIL into White, Asian, Mixed, Black, and Other. Smoking status is categorised in SAIL into Current Smoker, Former Smoker, Never Smoker, and Unknown [20]. In cases where women had multiple recorded statuses, the most recent status during or prior to pregnancy was selected.

The study start date of 13th April 2021 was selected because phase 2 of the vaccination program, which aimed to provide vaccinations to individuals aged 40 to 49, 30 to 39, and 18 to 29 years, commenced on this date. The inclusion criteria were currently pregnant women who had not received the vaccination or had one dose of vaccination before pregnancy, alive, known pregnant on the first day of follow-up, and aged 18 years or older. Pregnancies identified later in the study period were followed until delivery. The exclusion criteria were women who were fully vaccinated (i.e., two vaccinations) before pregnancy, those for whom it was not possible to determine the start date of pregnancy due to unavailability of the gestational age and initial assessment dates in their records or those with miscarriage or stillbirth outcomes. Currently, within SAIL researchers are unable to account for terminations as these are classed as sensitive data and not currently accessible for research purposes.

### Calculating pregnancy start date

Pregnancy start dates were calculated from the following sources:

For pregnancies identified from the NCCHD and MIDS data, the pregnancy start dates were calculated based on the gestational age and the week of birth data items available in these data sources. In cases where gestational age is missing, a value of 40 weeks was applied as the majority of those with missing data (92%) had birth weights suggestive of full-term infants. Thus, the pregnancy start date (last menstrual period) was simply calculated by subtracting the gestational age at birth (in weeks) from the week of birth. Pregnancies identified from both data sources were compared/matched and duplicate records were removed.

For pregnancies identified from the WLGP data, all pregnant women with a pregnancy code and event date that occurred during the study period were extracted (Supplementary Table 1: Read codes (v2)). For those identified from the hospital admissions data (PEDW), all women with a pregnancy diagnosis code and an attendance date occurring during the study period were also extracted (Supplementary Table 2: ICD-10 codes). Identified cases from both the WLGP and PEDW were separately matched to those identified from the NCCHD and MIDS data to include only those who are still pregnant. Furthermore, the identified cases from both resources were further matched to remove duplicates and then linked to the initial assessment-related data items in the MIDS data. The gestational age in weeks and initial assessment data items are available in order to calculate the pregnancy start date. In cases where multiple records were found per pregnant woman, only the first occurring record between the study dates of interest was selected. The pregnancy start date for every successfully linked case was then calculated by subtracting the gestational age from the initial assessment date.

### Multimorbidity in pregnancy

Multimorbidity was defined by the presence of two or more long-term health conditions, which can include defined physical and mental health conditions [8]. Long-term health conditions are those that generally last a year or longer and have a significant impact on a person’s life [9]. Four long-term health conditions including depression, diabetes, asthma, and cardiovascular were selected on the following basis: (1) prevalence; (2) potential to impact vaccine uptake; and (3) recorded in the study datasets. These conditions were aggregated to generate a new multimorbidity variable with two distinct categories: Multimorbid and Non-multimorbid. The multimorbid category comprises those with two or more health conditions, while the non-multimorbid comprises healthy individuals together with those with only one health condition. Read codes for depression, diabetes, asthma, and cardiovascular can be found in Supplementary Tables 3 to 6 respectively. ICD-10 codes for the same conditions can be found in Supplementary Tables 7 to 10 respectively.

### Statistical analysis

Kaplan-Meier survival analysis was employed to examine time to vaccination by depression, diabetes, asthma, and cardiovascular diseases independently, by multimorbidity, as well as by smoking status censored at the delivery, death, or moved out of Wales while pregnant. The Log Rank test was used to determine if there were differences in the survival distributions of vaccine uptake times within the diseases independently, multimorbidity, and smoking status. Differences were reported in median times (MD) with 95% confidence intervals and significance level accepted at p < 0.05. Multivariate Cox regression hazard models were utilised to examine the impact of depression, diabetes, asthma, and cardiovascular diseases on vaccine uptake independently with age group, ethnic group, area of deprivation, and smoking status incorporated into the model, as well as the impact of multimorbidity, age group, ethnic group, area of deprivation, and smoking status on vaccine uptake, reporting hazard ratios (HR) with 95% confidence intervals and significance level accepted at p < 0.05. Bootstrapping internal validation was conducted to assess the performance of the model, reporting bootstrapped Beta coefficients (B), standard error, 95% confidence intervals, and significance level accepted at p < 0.05. The reference groups were those without multimorbidity, never smokers, aged 25–29, white ethnic group, and those living in the most affluent area. The data handling and preparation for the descriptive statistics, survival analysis, and Cox proportional hazard modelling were performed in an SQL IBM DB2 database within the SAIL Databank utilising Eclipse software. Final data preparation specific to these analyses, such as setting the reference groups was performed in IBM SPSS Statistics 28. Descriptive statistics, Survival, and Cox regression analyses were performed in SPSS.

## Results

**Fig. 1:**
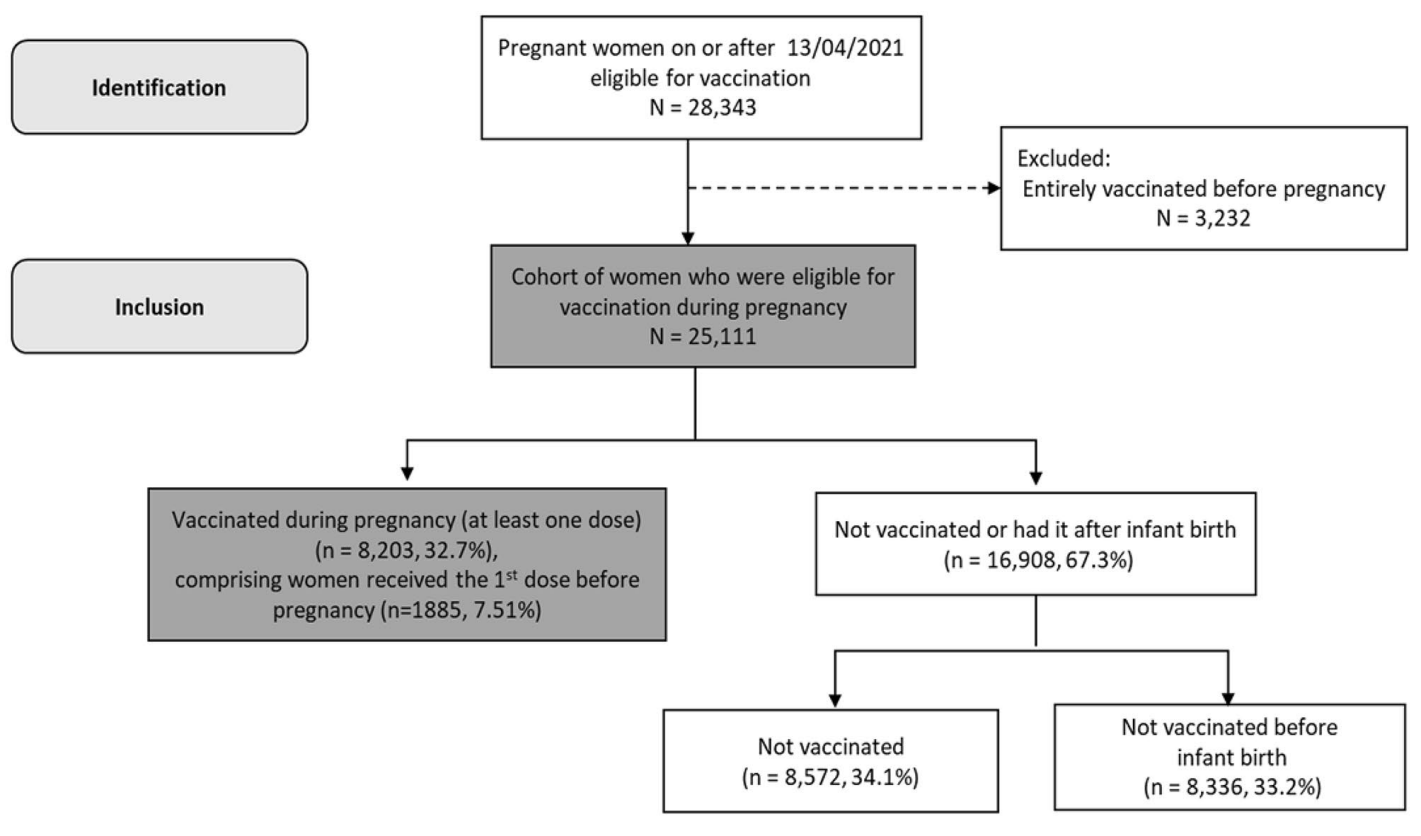
Cohort identification flowchart

A total of 28,343 pregnant women were identified from 13th April 2021 through 31st December 2021. After excluding women who were fully vaccinated prior to becoming pregnant (n = 3,232), the cohort consisted of 25,111 pregnant women accounting for those who were censored due to death (n = 106) or relocation outside of Wales during pregnancy (n = 358). Following up with those women, their records were linked to the COVID-19 vaccination data through and including 31st December 2021. Over the study period, 8,203 (32.7%) of pregnant women received one or more doses of the COVID-19 vaccination which comprises those who received the first dose before pregnancy, 8,572 (34.1%) remained unvaccinated, and 8,336 (33.2%) received the vaccine postpartum (Fig. 1 illustrates the participants of the cohort). The majority of the women were between the ages of 25–29 and 30–39 (29.7% and 48.4% respectively). 77.8% were White. 23.3% were living in the most deprived areas, while 14.4% were living in the least deprived areas. 29.5% of women were diagnosed with depression, 5.8% had diabetes, 23.9% had Asthma, 3.3% had cardiovascular, and 12.9% were multimorbid (i.e., Had two or more health conditions) (Table 1).

**Table 1:** Descriptive summaries of the pregnant women eligible for vaccination

		N	%
**Age group**	18–24	4,664	18.6
	25–29	7,447	29.7
	30–39	12,143	48.4
	40–50	857	3.4
**Ethnic group**	White^1^	19,547	77.8
	Asian^2^	902	3.6
	Mixed^3^	316	1.3
	Black^4^	440	1.8
	Other^5^	571	2.3
	Unknown	3,335	13.3
**WIMD Quintile 2019**	1st (Most deprived)	5,840	23.3
	2nd	4,795	19.1
	3rd	4,157	16.6
	4th	3,804	15.1
	5th (Least deprived)	3,626	14.4
	Unknown	2,889	11.5
**Smoking Status**	Current smoker	4,441	17.1
	Former smoker	3,150	12.5
	Never smoker	12,418	49.5
	Unknown	5,102	20.3
**Depression**	Yes	7,419	29.5
	No	17,692	70.5
**Diabetes**	Yes	1,468	5.8
	No	23,643	94.2
**Asthma**	Yes	5,999	23.9
	No	19,112	76.1
**Cardiovascular**	Yes	826	3.3
	No	24,285	96.7
**Multimorbidity**	Yes	3,247	12.9
	No	21,864	87.1

^1^Comprises of Any White Background, Gypsy or Irish Traveller

^2^Comprises of Bangladeshi, Pakistani, Indian, Any Other Asian Background

^3^Comprises of Any Other Mixed Background, White and Asian, White and Black African, White and Black Caribbean, Any Other Mixed/Multiple Ethnic Background

^4^Comprises of African, Any Other Black Background, Caribbean

^5^Comprises of Any Other Ethnic Group, Arab, Chinese

### Examining the impact of multimorbidity, smoking status, and demographics on vaccine uptake

Results revealed a statistically significant association between multimorbidity and vaccine uptake (HR = 1.12, 95% CI 1.04 to 1.19, p = 0.001). This indicates that women living with multimorbidity were 1.12 times more likely to have the vaccine compared to those with no multimorbidity (Table 2). Women who had depression were slightly but significantly more likely to have the vaccine compared to those without depression (HR = 1.08, 95% CI 1.03 to 1.14, p = 0.002) (supplementary Table 12). Diabetes, asthma, and cardiovascular diseases were not associated with vaccine uptakes (HR = 1.01, 95% CI 0.93 to 1.11; HR = 1.05, 95% CI 0.99 to 1.10; HR = 1.06, 95% CI 0.94 to 1.19 respectively) with p > 0.05 for all (Supplementary Tables 13–15). Vaccine uptakes were significantly lower among both Current Smokers and Former Smokers compared to Never Smokers (HR = 0.87, 95% CI 0.81 to 0.94, p < 0.001) and (HR = 0.92, 95% CI 0.85 to 0.98, p = 0.015) respectively. Those aged 40–50 were 1.33 times more likely to have the vaccine compared to those aged 25–29 (HR = 1.33, 95% CI 1.18 to 1.49, p < 0.001), also those aged 30–39 were 1.17 times more likely to have the vaccine compared to those aged 25–29 (HR = 1.17, 95% CI 1.12 to 1.24, p < 0.001), indicating a significant positive association between older women aged > 30 and vaccine uptake. It was also observed that the vaccine uptake was lower among those living in the most deprived areas compared to those living in the most affluent areas (HR = 0.89, 95% CI 0.83 to 0.96, p = 0.002).

**Table 2:** Cox Regression analysis of factors associated with vaccination uptake among pregnant women eligible for vaccination, adjusted analysis

Characteristic		HR^1^ (95% CI^2^)	*P* value^3^
**Age group**	25–29	Reference	
	18–24	0.99 (0.92–1.07)	0.788
	30–39	1.17 (1.12–1.24)	< 0.001
	40–50	1.33 (1.18–1.49)	< 0.001
**Ethnic groups**	White	Reference	
	Asian	1.09 (0.97–1.22)	0.140
	Mixed	1.02 (0.81–1.28)	0.899
	Black	1.06 (0.87–1.29)	0.577
	Other	1.16 (1.00–1.33)	0.050
	Unknown	0.94 (0.87–1.01)	0.067
**WIMD quintile 2019**	5th (Least deprived)	Reference	
	4^th^	0.90 (0.84–0.97)	0.005
	3^rd^	0.82 (0.76–0.88)	< 0.001
	2^nd^	0.92 (0.85–0.99)	0.018
	1st (Most deprived)	0.89 (0.83–0.96)	0.002
**Smoking status**	Never Smoker	Reference	
	Former Smoker	0.92 (0.85–0.98)	0.015
	Current Smoker	0.87 (0.81–0.94)	< 0.001
	Unknown	1.09 (1.03–1.15)	0.003
**Depression**	No	Reference	
	Yes	1.08 (1.03–1.14)	0.002
**Diabetes**	No	Reference	
	Yes	1.01 (0.93–1.11)	0.761
**Asthma**	No	Reference	
	Yes	1.05 (0.99– 1.10)	0.073
**Cardiovascular**	No	Reference	
	Yes	1.06 (0.94– 1.19)	0.367
**Multimorbidity**	No Multimorbidity	Reference	
	Multimorbidity	1.12 (1.04–1.19)	0.001

^1^Hazard Ratio, ^2^Confidence Interval (95%), ^3^significance level accepted at < 0.05

The models were internally validated to estimate their performance more accurately. Bootstrap resampling started with fitting the regression model in a bootstrap sample of 1000 random samples, which were generated with replacement from the original dataset. Bootstrapping analyses were performed on each random sample, and beta coefficient, standard error, and 95% Bias corrected accelerated (BCa) confidence intervals for the primary findings were generated. Bootstrapping estimated the internal validity, providing stable estimates with low bootstrapped bias, low standard errors, and robust confidence intervals for both the multimorbidity and depression models (Supplementary Tables 16 and 17 respectively).

### Examining time to vaccination in pregnancy

**Fig. 2:**
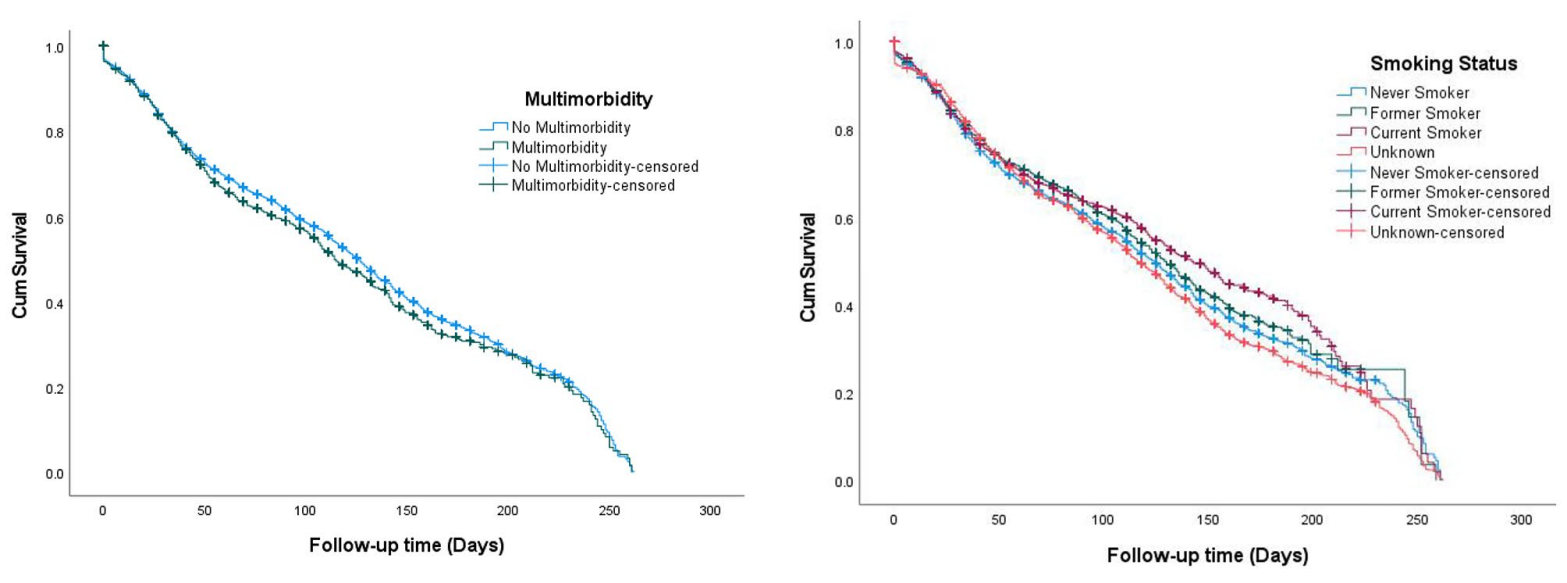
2a. Time to vaccine uptake by Multimorbidity. 2b. Time to vaccine uptake by Smoking status

Kaplan-Meier survival analysis indicates that pregnant women living with multimorbidity had a median time to vaccine uptake of 114 days (95% CI 106.6 to 121.4). This was lower than those with the absence of multimorbidity, which had a median time to vaccine uptake of 126 days (95% CI 123.4 to 128.6) (Fig. 2a). However, other survival analyses were also conducted to estimate the time to vaccination for depression, diabetes, asthma, and cardiovascular diseases independently. The median times to vaccine uptake were not significantly different between the different groups for all the diseases (Supplementary Table 11). Current Smoker women had a median time to vaccine uptake of 142 days (95% CI 132.6 to 151.4). This was longer than Never Smokers and Former Smokers, which had median times to vaccine uptake of 124 days (95% CI 120.9 to 127.1) and 129 days (95% CI 121.7 to 136.3) respectively (Fig. 2b).

## Discussion

This study investigated the impact of multimorbidity and smoking status on vaccine uptake as well as the impact of depression, diabetes, asthma, and cardiovascular diseases independently during pregnancy in Wales. Women with depression were slightly but significantly more likely to have the vaccine compared to those without depression. Those living with multimorbidity were also more likely to have the vaccine compared to those living with the absence of multimorbidity, indicating a statistically significant positive association with vaccine uptake. Vaccine uptake was lower among those who currently smoke and those former smokers compared to those who have never smoked. Results also revealed a significant positive association between older women and vaccine uptake. Specifically, women aged 30 or above exhibited a higher likelihood of receiving the vaccine compared to younger women. Uptake was lower among those living in the most deprived areas compared to those living in the most affluent areas. These findings may help to generate and tailor vaccine strategies to the populations who are more vaccine hesitant. The findings of the study complement previous studies of vaccine hesitancy. The presence of one or more chronic conditions has been found to be associated with the willingness to receive the COVID-19 vaccine [11]; this study supported that those with multimorbidity are more likely to accept the vaccine than non-multimorbid individuals. In previous studies, current smokers reported significantly greater mistrust of vaccines and any benefits and were more worried about future outcomes [13]. Current smokers were more likely to be unwilling to have the vaccine; 21.5% compared to 11.6% of never smokers, (OR = 2.12, 95% CI 1.91–2.34, p < 0.001) and compared to 14.7% of former smokers (OR = 1.53, 95% CI 1.37–1.71, p < 0.001) [13]. The findings of the study also indicated that current and former smokers were less likely to accept the COVID-19 vaccine. The ‘Understanding Society’ COVID-19 survey asked participants (n = 12,035) their likelihood of vaccine uptake and reason for hesitancy. Vaccine hesitancy was high in Black (71.8%) (OR = 13.42, 95% CI 6.86–26.24, p = < 0.05) and Pakistani/Bangladeshi (42.3%) (OR = 2.54, 95% CI 1.19–5.44, p = < 0.05) ethnic groups compared to White British/Irish [15]. Conversely, this study revealed that the Other ethnic group were slightly but marginally significantly less likely to be hesitant toward COVID-19 vaccination during pregnancy when compared to White women of British/Gypsy/Irish descent (HR = 1.16, 95% CI 1.00 to 1.33, p = 0.05). On the other hand, the study found no statistically significant difference between White and Black ethnic groups (HR = 1.06, 95% CI 0.87 to 1.29, p = 0.58) or White and Asian ethnic groups (HR = 1.09, 95% CI 0.97 to 1.22, p = 0.14). The results also revealed that those living in more deprived areas in Wales were less likely to accept the vaccine. Similar results were found with adults living in the most deprived areas of England (based on the Index of Multiple Deprivation) were more likely to report vaccine hesitancy (8%) than adults living in the least deprived areas (2%) [16].

### Strengths and limitations

The study has several strengths; it utilises primary and secondary health care data for pregnant women in Wales including the maternity and child health data, it gives a national perspective of COVID-19 vaccine hesitancy, making the findings generalizable due to its total population cohort. Bootstrapping internal validation was performed to estimate the performance of the models with increased accuracy. The study had specific limitations, including the absence of data pertaining to the trimester during which pregnant women received the vaccine. Existing literature indicates that pregnant women in the first trimester expressed higher acceptance of COVID-19 vaccination in comparison to those in the second and third trimesters [14]. Additionally, another constraint pertains to the exclusion of calendar time as a factor in assessing vaccination uptake and its potential confounding influence. This omission arose due to the unavailability of this factor in the study dataset. The study excluded miscarriage and stillbirth outcomes as these are classed by SAIL as sensitive data and are not currently accessible for research purposes.

It is crucial to recognise that certain potential cofounders, not available in the electronic health records, need to be considered. Confounding factors previously associated with vaccine hesitancy include convenience factors, perceived risk of vaccination, and social media influence [21]. Moreover, an individual’s mental health may impact their decision to receive or decline vaccination. Previous research has indicated that individuals with mental health conditions, particularly emotional disorders such as anxiety and phobias, may have an increased risk of vaccine hesitancy [22]. These factors can influence an individual’s vaccination choice. Future research could explore these factors through qualitative analysis to gain deeper insights into the underlying reasons contributing to vaccine hesitancy.

## Conclusion

In conclusion, it is critical to develop tailored strategies to increase the acceptance rates of the COVID-19 vaccine and decrease hesitancy. A more targeted approach to vaccinations may need to be addressed to reach certain groups such as younger people, smokers and former smokers, healthy individuals, and those living in higher deprivation level areas. Future research endeavours could incorporate a qualitative analysis of these factors to explore the underlying rationales contributing to the impact of these factors on vaccine hesitancy. Encouraging vulnerable populations including pregnant women is a priority moving forward.

### Electronic supplementary material

Below is the link to the electronic supplementary material.

Supplementary Material 1

## Data Availability

Data that support the findings of this study are available from SAIL, however, restrictions apply to the availability of these data, which were used under license for the current study, and so are not publicly available. Data are however available from the corresponding author (Mohamed Mhereeg) upon reasonable request and with permission of SAIL.

## List of abbreviations

EHR: Electronic Health Records
SAIL: Secure Anonymised Information Linkage
HR: Hazard Ratios
SAGE: The Scientific Advisory Group for Emergencies
JCVI: The UK’s Joint Committee on Vaccination and Immunisation
GP: General Practitioners
WLGP: Wales Longitudinal General Practice
PEDW: Patient Episode Database for Wales
OPDW: Outpatient Database for Wales
NCCH: National Community Child Health
MIDS: Maternal Indicators Dataset
CVVD: COVID-19 Vaccination Data
WDSD: Welsh Demographic Service Dataset
WIMD: Welsh Index for Multiple Deprivation
MD: Median Times
SQL: Structured Query Language
SPSS: Statistical Package for Social Sciences
WHO: The World Health Organisation
IGRP: Information Governance Review Panel
HDR: Health Data Research
BHF: British Heart Foundation
UK: United Kingdom
